# Surrogate Clinical End Points in Studies of APOL1-Associated Kidney Disease: Just in Time

**DOI:** 10.2215/CJN.0000000628

**Published:** 2024-12-16

**Authors:** Akinlolu O. Ojo

**Affiliations:** University of Kansas School of Medicine, Kansas City, Kansas

**Keywords:** apolipoprotein L1 (APOL1)

Apolipoprotein L1 (*APOL1*) high-risk genotype is a well-established cause of primary glomerular disease and a progression factor in CKD associated with other etiologies such as hypertension, diabetes mellitus, sickle cell disease, and SLE in people of recent African ancestry.^1,2^ *APOL1* kidney disease, *APOL1*-associated kidney disease, and *APOL1*-mediated kidney disease are umbrella terms encompassing several kidney disease phenotypes with differing etiologies and contributory factors, all with a common element of the presence of *APOL1* G1 and/or G2 alleles. The large effect size of the *APOL1* high-risk genotype on the development and progression of kidney disease and its remarkably high population attributable risk are two main factors that have fueled hundreds of epidemiologic, mechanistic, and clinical studies on *APOL1*-associated kidney disease.^3,4^ Because of the significance of *APOL1*-associated kidney disease as the major driver of racial disparities in CKD and its treatment, it took less than a decade (a notably relatively short time) between the identification of the roles of *APOL1* kidney risk variants and the initiation of clinical development programs, including therapeutic registration clinical trials of novel agents.^5,6^ Thus, the study by Rosenberg *et al.*^7^ is well timed, urgently needed, and likely to influence the design of clinical trials of therapeutic agents to treat *APOL1*-associated kidney disease.

The three prospective cohort studies in the study by Rosenberg *et al.*^7^ (African American Study of Kidney Disease and Hypertension [AASK], Chronic Renal Insufficiency Cohort [CRIC], and Atherosclerosis Risk in Communities) are landmark studies that have collectively contributed immensely to the understanding and management of CKD with many findings that have changed clinical practice. The three cohort studies (AASK was a clinical trial with a cohort follow-up) were designed to address different but related questions; hence, their study populations and follow-up schemes were dissimilar. The existence of the United States Renal Data System and the fact that each of the cohort studies measured kidney function, kidney disease, and cardiovascular disease (CVD) outcomes created a powerful research resource that was innovatively used by Rosenberg *et al.*^7^ to conduct the landmark study reported in the current issue of *CJASN*.^7^

Rosenberg and colleagues in their study examined four surrogate clinical end points (3-year 30% and 40% decline in GFR, doubling of urine protein–creatinine ratio, and >3 ml/min per 1.73 m^2^ per year decline in GFR) to test the hypothesis that these surrogate end points would predict long-term clinical outcomes independent of *APOL1* genotype risk status and therefore be eligible candidates as clinical trial end points. Rosenberg *et al.* noted that only two of the four surrogate end points in their study have been accepted by the major regulatory agencies as valid end points for clinical trials in CKD and glomerular diseases. The authors did not provide a rationale why the two other US Food and Drug Administration–accepted surrogate end points (between-arm eGFR slope reduction of >0.5–1.0 ml/min per 1.73 m^2^ per year—accepted validated end point and a between-arm reduction of ≥30% in the urine albumin–creatinine ratio—reasonably likely end point) were not studied although the data to derive these two additional end points were likely to have been available in both the AASK and CRIC studies. Instead, they were substituted without explanation. Notwithstanding, the study by Rosenberg *et al*. is innovative as the first to test validated US Food and Drug Administration–accepted surrogate clinical end points in studies of APOL1-associated kidney disease.

The immediate question of high importance is “Should the four surrogate end points in the Rosenberg study be adopted as valid surrogate clinical trial end points by clinical trialists and the regulatory agencies in studies of *APOL1*-associated kidney disease?” In the judgment of this commentator, the answer to the foregoing question is a qualified yes. The reasons for accepting the surrogate clinical end points in this study as valid are the following. First, the study is based on a large sample size (4678 participants) among whom a high number of surrogate outcome events (1,325) were recorded—479, 272, and 574 outcome events for 30% GFR decline, 40% GFR decline, and >3 ml/min per 1.73 m^2^ per year decline in GFR, respectively. Second, the study was based on three cohort studies, each with a prospective design that has long duration of follow-up during which the phenotype of the participants was comprehensively characterized at both baseline and during follow-up. Third, the findings were consistent across the three cohorts when they were analyzed separately. Finally, the surrogate end points predicted long-term clinical outcomes without an interactive effect of the *APOL1* genotype risk status. A qualified yes is warranted because the three cohort studies were heterogeneous as confirmed by high I^2^ for GFR decline of 40% and doubling of the urine protein–creatinine ratio in the statistical test of heterogeneity. It should be noted that qualifying the yes answer to the question posed *vide supra* on the basis of a statistically insignificant test of heterogeneity alone may be excessively strict because heterogeneity in the study population is not always damaging to validity in that in some cases, heterogeneity in the study arms does not compromise the generalizability of treatment effect.^8^

The use of surrogate clinical end points as proof of therapeutic efficacy harbors huge ramifications, hence the need for validation studies to raise the confidence level in the surrogate clinical end points. Ergo, the question arises, “Should we wait for validation studies of the Rosenberg *et al.* findings before deploying their four surrogate end points in the in clinical trials of *APOL1*-associated kidney disease?” The answer to this question is an unqualified no. First, there is no other extant cohort with a large sample of Black study participants with CKD that could serve as a validation cohort. Parenthetically, the lack of any other such extant cohorts or study populations is a lamentable reflection of the nation's research priorities for a glaring omission in our research portfolio—there have been no adequately powered, focused studies of kidney disease in Black individuals since the AASK clinical trial was completed two decades ago. This is despite the fact that, for more than 50 years since we have been collecting data on kidney failure, Black individuals continue to suffer from kidney failure at a rate that is three times higher than that of non-Hispanic White individuals. Second, establishing a new validation cohort will take a decade or more to accrue an adequate sample size and accumulate sufficient follow-up times. Finally, the heterogeneity problem of combining the three different prospective cohorts in the current study is only a notable design feature and not an invalidating methodologic concern.

An essential attribute of a valid surrogate clinical end point is that it should predict long-term clinical outcomes reliably. In this study, kidney failure (with need for KRT), CVD events, and mortality were considered as long-term clinical outcomes. Conditional Cox regression model (conditioned on kidney disease-free survival at the 3-year follow-up) showed that the eGFR surrogate end points were reliably predictive of kidney failure, CVD, and death. That the predictive power of the surrogate clinical end points was not modified by the *APOL1* genotype risk status is an intrinsic measure of validity of the surrogate outcomes supporting their utility as clinical end points in trials in which the target of intervention is the APOL1 kidney risk genotype. However, the study used the Cox regression model which does not assume a hazard function opening a potential source of estimation bias. The study might have considered alternative parametric models with known hazard functions.^9,10^

Individuals with primary FSGS or other primary glomerular diseases caused by or associated with high *APOL1* risk genotype account for a very small fraction (<10%) of people of recent African ancestry with *APOL1*-associated kidney disease. Most individuals who are likely to benefit from *APOL1*-targeted therapeutic interventions have CKD associated with hypertension, diabetes mellitus, sickle cell disease, SLE, and other causes^1,2^—these are the same individuals enrolled in the CRIC, AASK, and Atherosclerosis Risk in Communities studies, thus making the study by Rosenberg *et al.* even more relevant and significant. Clinical trials of *APOL1*-targeted therapy in individuals like those in the study by Rosenberg *et al.* are highly likely to confer the greatest personal medical benefits and population health improvements in people of recent African ancestry worldwide. The world having waited too long, it cannot be soon enough for APOL1 late-stage clinical trials to commence in earnest globally especially now that Rosenberg and colleagues have given us the necessary tools both to evaluate therapeutic efficacy and to measure clinical outcomes in such clinical trials.
